# Comorbidity and therapeutic response of body dysmorphic disorder (BDD) in autism spectrum disorder (ASD)

**DOI:** 10.1192/j.eurpsy.2021.1113

**Published:** 2021-08-13

**Authors:** A. Alvarez Pedrero, A. González-Rodríguez, D. Garcia Pérez, L. Delgado, G.F. Fucho, I. Parra Uribe, S. Acebillo, J.A. Monreal, D. Palao Vidal, J. Labad

**Affiliations:** 1 Mental Health, Parc Taulí University Hospital. Autonomous University of Barcelona (UAB). I3PT, Sabadell, Spain; 2 Mental Health, Parc Taulí-University Hospital, Sabadell, Spain; 3 Mental Health, Parc Tauli University Hospital, Sabadell, Spain; 4 Mental Health, Hospital of Mataró. Consorci Sanitari del Maresme. CIBERSAM., Mataró, Spain

**Keywords:** body dysmorphic disorder, autism spectrum disorder, comorbidity, psychologicaL therapy

## Abstract

**Introduction:**

Autism spectrum disorder (ASD) is a neurodevelopmental disorder with a biological basis overlapped with obsessive compulsive disorders and body dysmorphic disorder (BDD). The combination of pharmacological treatment and psychological interventions have been considered the gold-standard

**Objectives:**

Our main objective was to present the case of a patient with ASD and comorbid BDD. As a second objective, we reviewed recent works on the common neurobiological substrate and therapeutic options for both conditions.

**Methods:**

(1)Clinical case: Patient with ASD and BDD, treated with fluoxetine 60 mg/day and aripiprazole 30 mg/day. (2)Non-systematic narrative review focused on neurobiological substrate and treatment of ASD and BDD. The electronic search was performed by the PubMed database (1990-2020) using the following key terms: “autism spectrum disorder”, “body dysmorphic disorder”, “dysmorphophobia”, “neurobiology”, “pharmacological treatment”, “psychological treatment” and “treatment”.

**Results:**

Our patient is a 31-year-old single male fulfilling DSM-5 criteria for ASD, diagnosed in childhood, and BDD. He received pharmacological treatment and CBT. He also verbalized having been concerned with his lips and mouth for the last 10 years. This discomfort leads to passive ideas of death. Review: All articles (n=4) supported the use of selective serotonin reuptake inhibitors (SSRIs) and CBT in this comorbidity. None of them reported the use of antipsychotics. Oone article described the use of Repetitive transcranial magnetic stimulation (rTMS) and oxytocin.

**Conclusions:**

ASD and BDD share the basis of corticostriatal circuits. ISRS and CBT may be effective in treatment. Other options (oxytocin or rTMS) should be further investigated. Examining this comorbidity could be useful for discovering possible endophenotypes.

